# An Overview of Nanostructured Lipid Carriers and its Application in Drug Delivery through Different Routes

**DOI:** 10.34172/apb.2023.056

**Published:** 2022-09-18

**Authors:** Shadab Khan, Ajay Sharma, Vikas Jain

**Affiliations:** Mahakal Institute of Pharmaceutical Studies, Ujjain, India.

**Keywords:** Drug delivery systems, Lipids, nanoparticles, Drug carriers, Permeability, Oral, Ophthalmic, Intransal

## Abstract

Nanostructured Lipid Carriers (NLC) are nano-sized colloidal drug delivery system that contains a lipid mixture consisting of both solid and liquid lipids in their core. This Lipid-Based Nanosystem is introduced as a biocompatible, non-toxic, and safe nano-drug delivery system as compared to polymeric or metallic nanoparticles. Due to its safety, stability, and high drug loading capacity compared to other lipid-based nanocarriers, NLC gained the attention of researchers to formulate safe and effective drug carriers. The ability to increase drug solubility and permeability while encapsulating the drug in a lipidic shell makes them an ideal carrier for drug delivery through difficult-to-achieve routes. Surface modification of NLC and the use of various additives result in drug targeting and increased residence time. With such qualities, NLCs can be used to treat a variety of diseases such as cancer, infections, neurodegenerative diseases, hypertension, diabetes, and pain management. This review focuses on the recent developments being made to deliver the drugs and genes through different routes via these nanocarriers. Here, we also discuss about historical background, structure, types of NLC and commonly employed techniques for manufacturing lipid-based nanocarriers.

## Introduction

 An effective drug delivery system is required to deliver drugs safely and effectively. Various carrier systems are being explored but there is a constant search for biocompatible, biodegradable, and stable carrier system with the ability to target specific organs. Fabricating materials for the carrier system are responsible for such properties. Lipids offered biocompatibility and biodegradability which is difficult to achieve with different materials. When these lipid-based systems are used as nanosized carriers they provide such properties which are difficult to attain in their bulk counterparts. Nanostructured lipid carriers (NLCs) are evolved as a novel drug delivery carrier.

 NLC has several advantages such as biocompatibility, biodegradability, non-immunogenicity, high drug loading capacity, better stability, controlled drug release, and easy preparation technique with scale-up ability. The advantages offered by these carriers make them ideal carrier for drug delivery. Potential cytotoxicity and chances of irritation due to surfactants are major drawbacks.^1^

 Scioli Montoto et al^2^ reviewed many articles regarding solid lipid nanoparticles (SLNs) and NLC by searching original publications in English in different databases. They found different therapeutic fields for which nanocarriers are prepared such as cancer, central nervous system (CNS) targeting (neurodegenerative diseases, psychosis, migraine, epilepsy, and depression), Antimicrobials, skin disorders, wounds, injuries, diabetes, antioxidants, non-steroidal anti-inflammatory drug (NSAID) and antihypertensives. The majority of formulations were developed to be delivered through the parenteral route followed by the oral route, cutaneous and transdermal route, nasal route, ocular route, and pulmonary and rectal route. This review focused on the different routes employed for the delivery of drugs, the historical background for the development of NLC along with structure and types of NLC, and different commonly employed manufacturing techniques.

## Need for nanostructured lipid carriers

 More than 50 years ago AD Bangham discovered “swollen phospholipids” as a model of the cell membrane, which was later termed liposome.^3^ From there on the spontaneous rearrangement of lipids to form nano colloidal particles have been constantly explored and evolved. Liposomes are established as drug delivery carriers in coming years with excellent properties to deliver drugs safely with increasing bioavailability and reducing toxicity.^4^ In 1995 with the first Food and Drug Administration (FDA) approval of liposomal doxorubicin a new era of lipid-based nanomedicine has been begun. Liposomes provide a safe and effective biodegradable platform for drug delivery. Vesicle-based lipid nanosystem did not meet expectations as a versatile drug carrier due to physiological stability, complex nature of nanomedicine, and cost of such medicine leading to a search for new lipid systems.^5^ Nanoemulsion is among the low-cost alternatives to the vesicle-based system but it is not effective in providing enough stability and safety to drugs.^6^

 Due to the shortcomings related to the earlier lipid-based nanosystem, a new class of lipid-based particulate systems emerged as an alternative by combining the advantage offered by liposome, nanoemulsion, and Polymer nanoparticles.^1^ In the early 1990s Müller et al independently developed SLNs with different methods, first-generation lipid nanoparticles as a cost-effective and versatile drug delivery system. These lipid-based nanosystems are mainly developed for cosmetics.^7,8^ SLN are solid lipid cores enclosed in lipidic and surfactant shells which can encapsulate the drug in a solid lipid matrix core. SLN has several advantages that make them suitable drug carriers for lipophilic drugs. They are physiologically more stable than other lipid-based nanosystems and contain biodegradable material for their fabrication, as well as a scalable fast, and effective manufacturing process.^9^ With a solid lipid core they can be stored for a long time in an aqueous condition which is impossible with liposomes.^10^ As solid lipid nanoparticle contains the organized solid lipid core, they have low drug loading capacity, which is a major drawback for efficient drug delivery. Initial burst release and stability issues on long-term storage are also a disadvantage of SLN such as the polymeric transition to crystalline form and drug leakage.^11^

**Figure 1:**
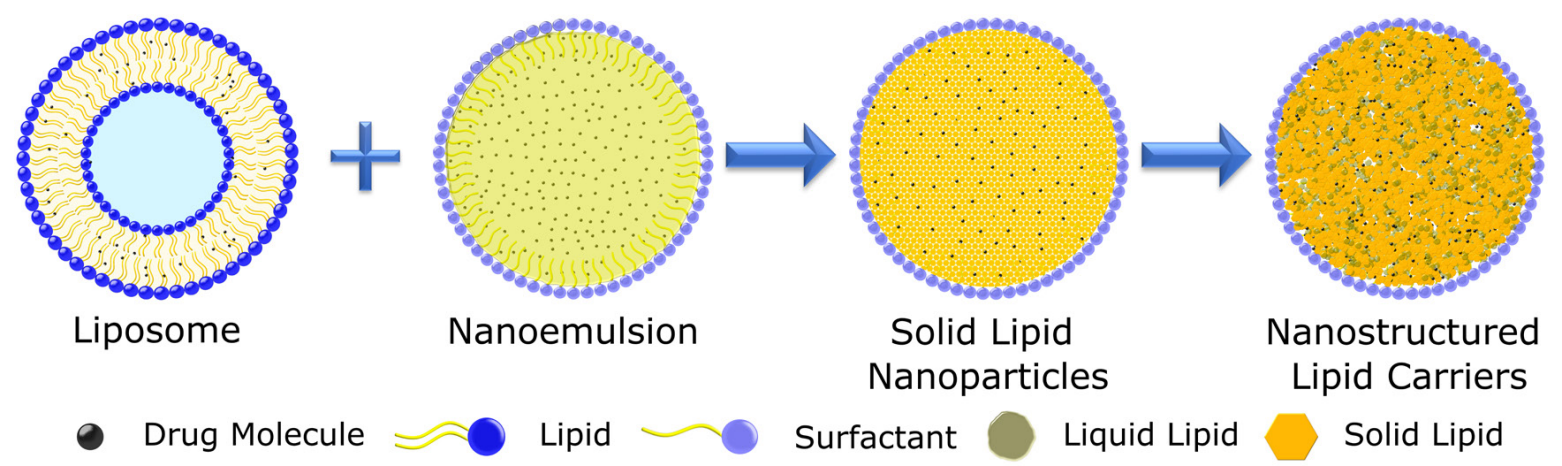
Evolution of lipid based nanocarriers

 In 1999 a new second-generation lipid nanoparticle emerged to overcome problems associated with the SLN. NLC is a nanocarrier that has advantages of previous lipidic nanoparticles along with a high drug loading capacity and increased stability than SLN.^12^ NLCs have made an entry into the cosmetic market in 2005 due to the properties they offered and now there are around 40 cosmetics products are there in the market. Due to the inherent qualities of NLC and their efficient encapsulation capacity this system has yet to hit the market as a drug delivery system.^13^ Figure 1 contains different types of lipids-based nanocarriers.

## Structure of nanostructured lipid carriers

 NLCs as the name suggest are nanosized multiparticulate system in the size range of 50 nm to 500 nm. The particle size distribution of NLC depends on nanoparticles’ manufacturing process and composition. Colloidal in nature this particle resembles the structure of SLN with the main variation being in the core of these nanoparticles.^14^ Unlike SLN which contains a solid lipid core in which lipid is arranged in a highly organized fashion, NLC contains lipid liquid along with a solid lipid core which forms an unorganized drug matrix. With this unorganized nature, more drugs can be loaded in the core in addition to stability issues encountered on long-term storage of SLN due to crystallization and drug expulsion is also rectified with NLC.^11^ NLC’s core is made up of solid lipid and liquid lipid in a 70:30 to 99.9:0.1 ratio, which distorts the NLC core. As the lipophilic molecule is more soluble in liquid lipid and the imperfection caused due to blends of lipid more space is available for drug incorporation in these nanocarriers.^15^ All materials used for the formulation of NLC should be considered generally regarded as safe (GRAS), as they should be non-toxic and biocompatible. Choice of lipid in NLC should also be made depending upon drug-lipid compatibility. Along with lipids which are used in a binary mix of solid and liquid lipids, one or a combination of surfactants are used within the range of 1.5% to 5% (w/v) to stabilize the nanosystem.^16^ Surfactant forms a coat around the NLC core. Choice of lipid and surfactant plays an important role in determining particle size and physicochemical properties of NLC. The type and number of surfactants is a crucial formulation parameter as using more than one surfactant results in less particle size and crystallinity than a single surfactant system. Two different surfactant results in a more stable system.^17^

**Figure 2:**
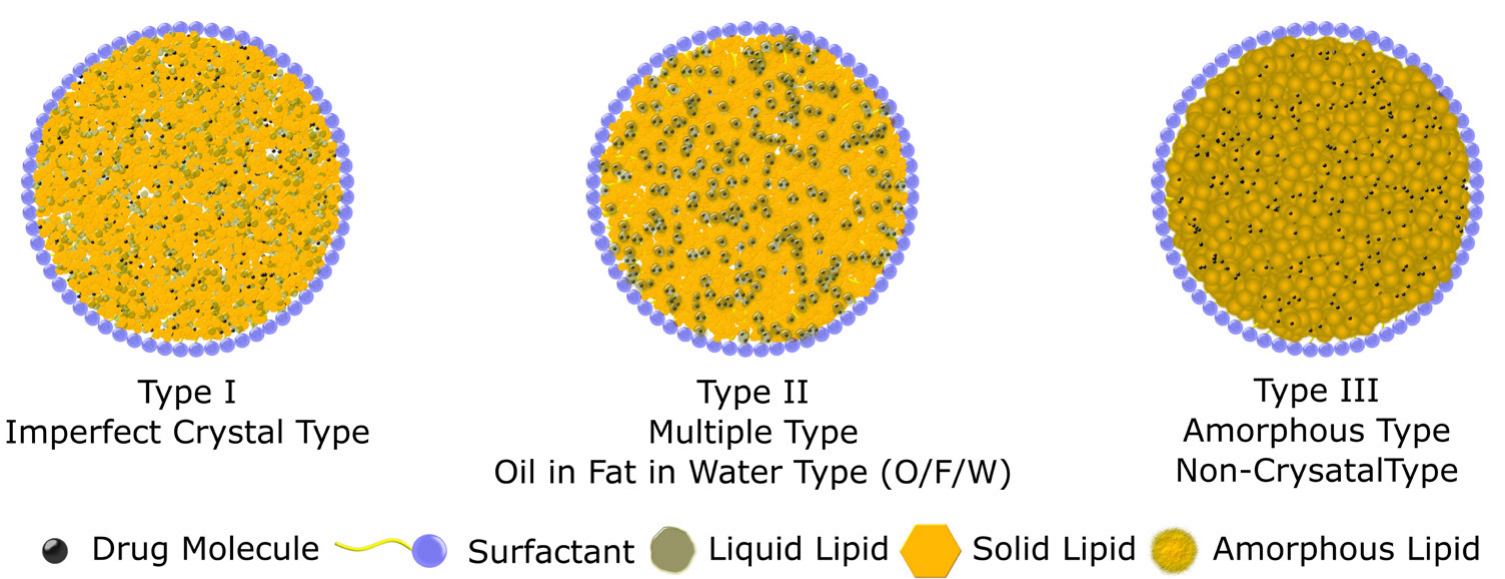
Types of nanostructured lipid carriers

 Variation in lipid content and formulation parameters results in a change in core structure and arrangement of solid lipid and liquid lipid. Three possible variations are classified by Muller et al as three different types of NLC. In the first type (imperfect crystal type), lipid content is low which results in deformation in solid lipid crystalline structure. Different fatty acid triglycerides can be used to modify the imperfection and structure of nanoparticles. Due to the fact, that lipophilic drugs are more soluble in liquid lipid, an increase in lipid concentration results in high drug incorporation. In the second type (Multiple types or oil-in-fat-in-water O/F/W carrier), they contain a high quantity of oil which results in a nano oil-based compartment within the particle resulting in a tiny packet of drug solubilized in liquid lipid. High liquid lipid solubility results in low drug leakage as well as slow drug release from this type of nanoparticles. The third type (Amorphous or non-crystalline type) is formed by blending solid and liquid lipid in a certain way to avoid crystallization of the core. This approach results in low drug expulsion due to the crystallization of the solid core.^18–20^ Figure 2 contains different categories of NLC.

## Methods of Preparation

 There have been several methods reported for preparing NLCs. Various preparation methods are as follows:

1. Hot high-pressure homogenization technique
2. Cold high-pressure homogenization technique
3. High speed/shear homogenization technique
4. Microemulsion
5. Solvent diffusion and evaporation technique
6. Hot melt extrusion
7. Solvent injection technique

## Hot high-pressure homogenization

**Figure 3:**
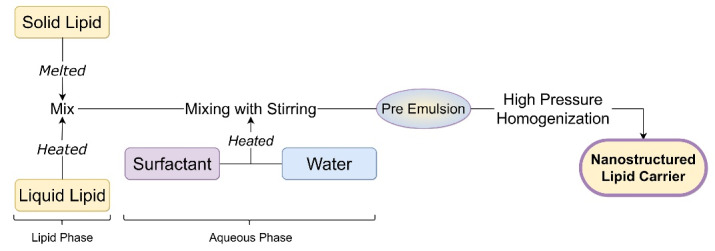
Hot high-pressure homogenization

 High-pressure homogenization is an energy-intensive and scalable technique to produce nano-sized colloidal systems (NLC, SLN, and nanoemulsions). It uses the top-down approach for the miniaturization of microemulsion particles to nano-size with the help of applied pressure.^21^ In this method, solid lipid was melted, then liquid lipid was added to form a heated lipid phase. Surfactants with or without cosurfactants are added to water to form an aqueous phase. Then the preheated lipid phase is mixed with the heated aqueous phase under constant stirring to create a microemulsion. This hot microemulsion is subjected to a high-pressure homogenizer for size reduction. Various homogenization cycles can be utilized based on the desired particle size. This nanoemulsion is cooled to be converted into NLC.^22,23^ Intermediate pressure (1000 bar) for long-time results in small particle size of NLC with less than 100 nm.^24^ This process is not preferred for drugs or materials which degrade at high temperatures. Figure 3 depicts systematic steps to formulate NLC.

## Cold high-pressure homogenization

Similar to hot high-pressure homogenization this process involves a mixture of lipid phase with a cold aqueous solution maintained at a temperature of approximately in the range of 2^∘^C to 6^∘^C with constant stirring. This coarse NLC suspension is homogenized with a high-pressure homogenizer at low temperatures. This process is suitable for drugs and materials which cannot be exposed to high temperatures.^25^

## High-speed homogenization

**Figure 4:**
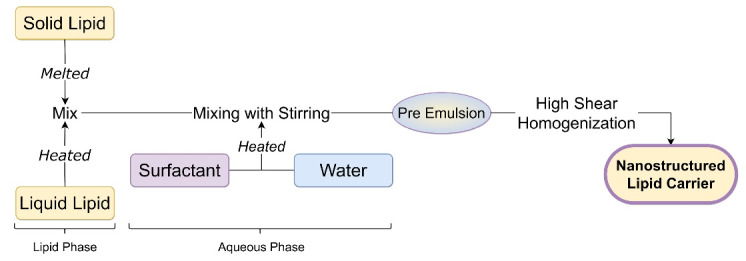
High speed homogenization

 This method of NLC preparation is identical to hot high-pressure homogenization. In this process, high pressure is replaced with a high shear rate. The lipid phase is prepared by mixing liquid lipid in melted solid lipid and the aqueous phase is prepared by mixing surfactant in water. This heated lipid and the aqueous phase are homogenized with a homogenizer at high rotation per minute (rpm) for a long time (10-30 minutes). The resulting solution is cooled at room temperature to form NLC.^26,27^ Speed of homogenization linearly affects the particle size of nanocarriers.^28^ Liquid nanoemulsion before cooling can also be sonicated for 5 minutes using an ultrasonic probe followed by cooling to further reduce the particle size of NLC.^29^ In some literature melt emulsification method was described in which the same technique was employed with low-speed homogenization and increased sonication time.^30,31^ Figure 4 shows a flowchart of steps required to formulate NLC through high-speed homogenization.

## Microemulsion

 In the microemulsion technique, the liquid lipid is added to the molten solid lipid. The resultant solution is mixed with an aqueous phase to form a microemulsion. This microemulsion is rapidly cooled with cold water to form NLC dispersion system. The difference in microemulsion and water dictates the particle size of NLC. This is a simple technique to prepare NLC but requires a high amount of surfactant and cosurfactant.^32^

## Solvent diffusion and evaporation technique

**Figure 5:**
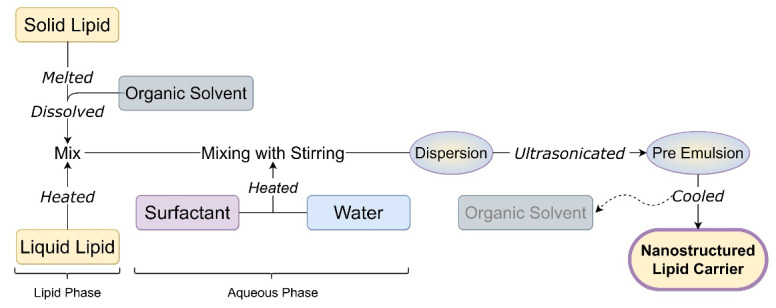
Solvent diffusion and evaporation technique

 In this technique, the liquid lipid is added to molten solid lipid which is dissolved in either a single or combination of organic solvents at high temperature. This lipid solution is then added to an aqueous solution containing surfactant with stirring This prepared dispersion is ultrasonicated to produce oil in water nanoemulsion which is cooled down with low stirring until the organic solvent is evaporated.^33^ This technique is low energy-intensive and avoids physical stress due to high pressure or shear, but due to the use of organic solvent, an additional step is required to remove the residual toxic solvent.^21^ Figure 5 shows solvent diffusion or solvent evaporation technique for the preparation of NLC.

## Hot melt extrusion technique

**Figure 6:**
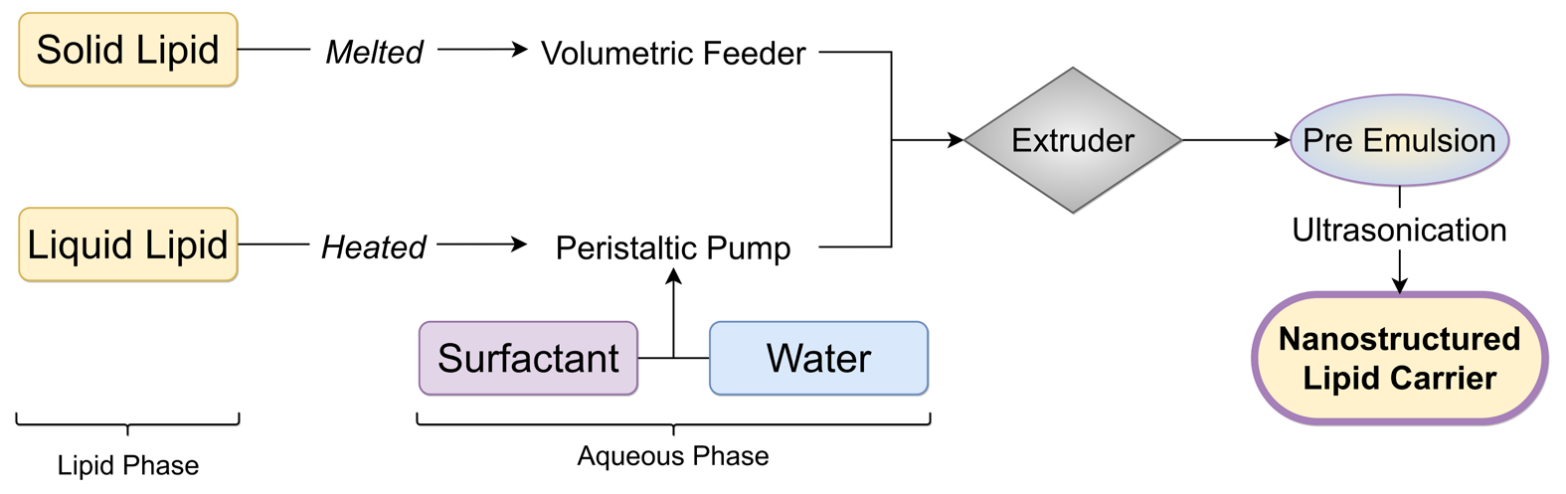
Hot melt extrusion technique

 The hot melt extrusion technique involves raw material pumping into a barrel, followed by sonication to obtain NLC. In this technique drug and solid lipid, the mixture was introduced in an extruder barrel using the volumetric feeder. Liquid lipid and aqueous solutions were added through a peristaltic pump at extrusion temperature. This mixture was extruded at component melt temperature to form a pre-emulsion. The resultant hot pre-emulsion is further sonicated to reduce NLC particle size.^34^ Figure 6 shows the steps to prepare NLC by hot melt extrusion technique.

## Solvent injection technique

**Figure 7:**
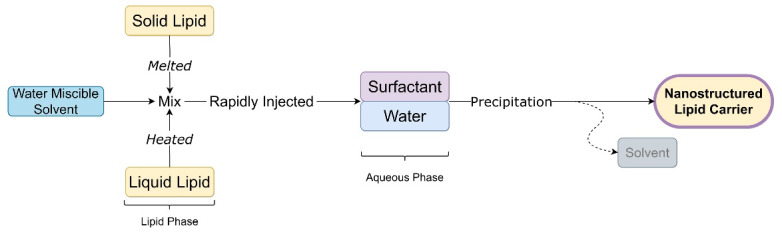
Solvent injection technique

 In this technique, the lipid phase is dissolved in a water-miscible solvent or their mixture with aid of heat to melt solid lipid. The resultant organic phase is rapidly injected into an aqueous phase containing surfactant or buffer solution with constant stirring. The solvent is diffused due to lipid precipitation and lipid nanocarrier formation. Particle size depends on solvent diffusion and emulsifier content.^21^ Figure 7 shows steps to prepare NLC by hot melt extrusion technique.

## Advantages of NLC

###  Drug delivery using NLC

 NLC have the inherent ability to deliver drugs safely and effectively through different routes. NLC can be employed to treat various ailments that require either localized drug delivery or targeted drug delivery. Along with the application of NLC for the delivery of drugs through different routes, these nanocarriers are also explored for the delivery of genetic material for gene therapy. Following are the examples of different routes employed for drug delivery using NLC.

###  Delivery through oral route

 The oral route is the most preferred route for drug delivery due to the convenience it provides. Drug delivery through the oral route is primarily considered by every formulator due to better patient compliance, easy administration without pain as in the parenteral route, and without assistance with low cost. But several hurdles make drug delivery through the route of choice difficult. Low solubility and permeability are the major challenges along with low stability in the harsh gastric environment leading to the delivery of drugs through a different route or use of carrier system. NLC provides one solution to deliver drugs efficiently through the oral route.^35^ There are several physiological challenges in the delivery of drug molecules through the oral route. pH gradient across the gastrointestinal tract (GIT) fluid is one of the major hurdles for drug stability as the drug molecule has to pass from the acidic environment in the stomach (pH 1.5-3.5) to the basic condition in the intestine (pH 5-8). Gastric and intestinal enzymes and secretions such as pepsin, bile, trypsin, etc. lead to further degradation of the drug in GIT. Along with this, sticky mucus covering the entire GIT makes the situation worse for the drug to penetrate this physiological barrier. Epithelial barrier, p-glycoprotein efflux pump in the gut wall, and first-pass metabolism make the oral delivery of most of the drug challenging task.^36,37^

 Lipid-based nanocarriers when delivered orally can be absorbed through various mechanisms such as transcellular transport, M cell-mediated transport, carrier-mediated transport, or by mucoadhesion. Unlike small and hydrophilic molecule which follows the paracellular pathway along the tight junction of enterocytes in the intestine, lipophilic drug and nanocarriers are transported through transcellular pathways. Transcellular absorption of the drug as well as nanocarriers is carried by endocytosis which can be occurred via phagocytosis through M cells. These M cells are the third most abundant cell type in the intestine and they are present in gut-associated lymphoid tissue (GALT) or Payer’s Patch. M cells do not contain a thick layer of mucus and are present to detect antigen and deliver molecules through the lymphatic system. Endocytosis is generally favored for naturally occurring molecules and the product made with the natural molecule, Thus NLC which contains lipid is favored for delivery of drugs through endocytosis.^36,38^ Gastric lipase and pancreatic lipase result in the breakdown of dietary triglyceride as well as triglyceride present in the lipid-based formulation, into di- and monoglycerides and fatty acids. The presence of exogenous lipid in the small intestine stimulates the secretion of bile salt, phospholipid, and cholesterol which arrange or make micelle or vesicular complex with exogenous lipid and facilitate absorption of these complexes.^39^ Attempts are being made to formulate NLC with bile salt such as sodium glycocholate, which increases many-fold increase in bioavailability.^40^ Formulation of NLC which forms a complex with bile salt and is uptake by the lymphatic system result in bypassing first-pass metabolism, which is a major hurdle for oral delivery of drugs with extensive hepatic metabolism.

 Another mechanism by which drug absorption will be increased is mucoadhesion. Mucoadhesion due to adherence of nanocarriers results in higher residence time of the drug in the GIT tract and an increase in time for the drug to be solubilized and absorbed through GI lining. Nanocarrier has a general tendency of adherence to the mucus layer on epithelial surface but a coating of the mucoadhesive layer further increases the mucoadhesion of NLC.^41^ Chitosan a natural mucoadhesive polysaccharide is frequently used to coat NLC particles, this coating results in double bioavailability than non-coated NLC.^42^ Various other materials are used to increase bioavailability and to sustain the release of the drug.

 As nearly half of the drugs being developed in recent years have low water solubility and the majority of nanomedicine that are being marketed or in the development phase are being delivered through the parenteral route. The parenteral route is not suitable for chronic conditions in which self-administration is preferred and conditions in which nontargeting is required such as hypertension, diabetes, infections, and chronic pain management. The oral route is also preferred in conditions that need local delivery of drugs in gastric conditions and drugs which are needed to be targeted to the liver.^43^ Table 1 contains different NLC formulations which are administered through oral route

**Table 1:** Drug delivery through oral route by NLC

**Drug**	**Purpose**	**Solid lipid**	**Liquid lipid**	**Surfactants**	**Formulation method**	**Outcome**	**Ref.**
Nisoldipine	Increase oral bioavailability of the hypertensive agent	Trimyristin	Oleic acid	Egg lecithin E 80 and poloxamer 188	Hot homogenization technique followed by ultrasonication	Drug loaded in NLC showed a prolonged drug release rate. Oral bioavailability increased by 2.46 and 1.09-fold in respect to suspension and SLN in rats.	^44^
Sulforaphane	Increase oral efficacy of anticancer agent	Precirol ATO 5	Vitamin E	Poloxamer 188 and tween 80	Melt emulsification method followed by ultrasonication	Optimized NLC show higher *ex vivo* drug permeability, improved cytotoxicity. Drug release is increased by more than 2-fold. 5-fold increase in relative bioavailability in comparison to suspension during *in vivo *studies in albino Wistar rats.	^45^
Insulin	Evaluate the impact of surfactant on peptide protection in gastric environment	Stearic acid	Peceol and oleic acid	Kolliphor RH 40, Brij L23, tegosoft PC41, and tegosolve 90 MB	Solvent diffusion method	PEG ether (Brij l23) shows the highest and PEG ester (tegosoft PC41 and tegosolve 90 MB) shows the lowest protection from GI proteases.	^46^
Rifabutin	Oral delivery of antimycobacterial drug in Crohn’s disease	Precirol ATO 5	Oleic acid	Tween 80 and epikuron 145	Hot high shear homogenization technique	*In vitro*, cellular studies show efficient macrophage uptake, increased permeation, and selective release in macrophages. NLC helped in achieving therapeutic concentration at a fast rate.	^26^
Quetiapine Fumarate	Increase bioavailability of antipsychotic a and reduce hepatic metabolism by lymphatic drug targeting	Precirol ATO 5	Oleic acid	Poloxamer 188 and phospholipon 90 G	Hot emulsification and ultrasonication technique	Pharmacokinetic study on rats shows an approximately 4-fold increase in AUC in rats than suspension. The experiment also shows lymphatic uptake of quetiapine fumarate in rats.	^47^
Lopinavir	Deliver antiretroviral drug orally	Compritol 888 ATO	Oleic acid	Poloxamer 188 and Tween 80	Hot high shear homogenization technique	Optimized NLC formulation shows higher cellular uptake in caco-2 cell line and 7-fold increase bioavailability in rats than drug suspension.	^27^
Cedrol	Increase bioavailability of antileishmanial drug	Compritol 888 ATO	Triolein	Pluronic F68 and soya lecithin	Hot melt emulsification ultrasonication method	*In vivo *studies show 2-fold increase in selectivity index (CC50/IC50) in both wild and drug-resistant leishmania and 2 to 3 fold and 3 to 5 fold increase of bioavailability in wild and drug-resistant leishmania respectively.	^48^
Adefovir dipivoxil	Oral delivery of antiviral drug for hepatitis B	Precirol ATO 5	Capmul MCM	Cremophor RH40 and pluronic F68	Solvent emulsification diffusion technique	*In *vivo, studies in Swiss albino mice demonstrate high uptake of the drug by liver cells, which will make NLC an appropriate liver targeting carrier.	^49^
Amphotericin B	Preparation of enteric-coated NLC for stability of antiviral drug in gastric pH	Glyceryl monostearate	Castor oil and labrasol	Tween 80, cremophor RH40 and PEG400	Hot high-pressure homogenization	Eudragit L100-55 coated nanoparticle shows a more than 4-fold increase in solubility than the free drug. Uncoated particle shows high drug solubility but is susceptible to acid degradation.	^22^
Fluconazole	Local delivery of antifungal drug for oral Candiasis by mucoadhesion	Stearic acid	Oleic acid	Pluronic F127 and lecithin	Emulsification and sonication technique	Optimized NLC were coated with chitosan results in sustained release of drug with prolonged antifungal effect due to mucoadhesion of NLC particles.	^50^
Berberine	Selenium coated NLC of antidiabetic phytomedicine for type 2 diabetes	Precirol ATO 5	Oleic acid	Tween 80	Hot melt dispersion and homogenization technique	Coating resulted in a 6.63-fold enhancement of oral bioavailability in rats than a solution with sustained release of the drug.	^51^

## Delivery through transdermal route

 Skin is a choice for drug delivery in many localized skin diseases and infections as well as delivery of drugs in a sustained and controlled fashion for the management of pain or wound healing. Skin being an easily accessible organ with a large surface area makes drug delivery through this route achievable without pain and undesired systemic side effects. Drug delivery through this route is mainly divided into dermal for localized effect and transdermal for deep skin penetration.^52^ Skin is a metabolically active organ with the main function of protecting the body from external dangers. Skin act as a barrier for external microorganisms, chemicals, or other molecules which try to enter the body and may create harm. This barrier function of the skin makes it difficult for therapeutically active molecules to directly enter the body.

 Anatomically skin is divided into the Epidermis, which contains an outer layer termed as stratum corneum with keratinized cells. And inner layer of the dermis and subcutaneous fat tissue. The outermost layer which is stratum corneum is the main barrier that is less permeable to avoid water and electrolyte loss. Brick and mortar-based multilayered arrangement of flattened corneocytes with extracellular lipid make penetration of large molecules impossible ( > 500 Da).^53^ Along with a less penetrable outer layer, other defensive features such as low skin pH, the presence of metabolizing enzymes make the situation difficult for many drugs.^54^

 Percutaneous absorption of a drug or other molecules is possible through pathways: First, the Transepidermal pathway includes diffusion of lipophilic solute through intercellular lipid domains or intracellular permeation of solute through imperfections in corneocytes. Second, the transappendageal pathway includes penetration of solute through the shunt pathway created by the hair follicles and sweat glands.^55^ As the transepidermal pathway favors drugs through the intercellular lipid domain present in the skin, lipophilic drug or carrier systems that have lipoidal properties are extensively explored. Conventional liposomes fail to permeate the skin barrier, deformable liposomes or flexible liposomes are being explored.^54^ SLNs are initially explored for cosmetic and pharmaceutical dermal applications. Second generation NLC with higher drug loading capacity and stability proved a carrier system for cosmetic preparation and many finished cosmetic preparations are marketed all over the world. Drug delivery by NLC through the skin has yet to reach its full potential.^56^

 NLCs-based topical formulations result in deeper skin penetration and sustained drug release with less systemic side effects and skin irritation.^57–60^ Various skin permeation enhancers can also be used to increase penetration of NLC in the skin.^61^ NLC also helps in decreasing wound healing time by delivering drugs at a sustained rate.^58,62^ NLC enhances the drug delivery through increased skin permeation due to its smaller size. NLC due to the occlusive nature of small size particle increase hydration in the skin layer and elasticity which result in better drug permeation because of less water evaporation from the skin surface. NLC also provides stability and protection to a drug molecule. Deeper penetration of NLC into the skin layer results in slow drug release which gave a prolonged effect with less frequent application.^8^ Table 2 contains various examples of NLC which are delivered through the transdermal route.

**Table 2:** Drug delivery through transdermal route by NLC

**Drug**	**Purpose**	**Solid lipid**	**Liquid lipid**	**Surfactant**	**Formulation method**	**Outcome**	**Ref.**
Tacrolimus	Immuno-suppressive agent transdermal delivery for atopic dermatitis	Glyceryl palmitostearate	Propylene glycol monocaprylate	Lecithin and Polysorbate 80	Hot high-pressure homogenization technique	NLC shows deeper penetration than nanoemulsion, with double the amount of drug in porcine ear hair follicle than nanoemulsion and approximately 4-fold increase from marketed preparation.	^57^
Recombinant human thrombomodulin	Angiogenesis factor loaded NLC for diabetic wound healing	Precirol ATO 5	Miglyol 812	Poloxamer 188	Hot homogenization technique	Sustained drug release is seen in comparison to solution. Better chronic wound healing and reduce systemic concentration results in less carcinogenic effect due to human growth factor.	^58^
Celastrol and Indomethacin	Treatment of rheumatoid arthritis and reduced systemic side effects	Precirol ATO 5	Labrasol ALF	Cremophor RH 40	Hot homogenization technique	NLC showed sustained drug release due to deep penetration by trans follicular pathway and small size. Cytokine’s expression and inflammatory response is also reduced in NLC based gel	^59^
Capsaicin	Local delivery for chronic pain management	Glyceryl monostearate	Cetyl alcohol	Isopropyl myristate	High shear homogenization followed by high pressure homogenization	Chilli extract loaded NLC gel shows more sustained drug release with less irritation suitable for chronic pain management	^60^
Pranoprofen	Topical delivery of NSAID for analgesic effect	Precirol ATO 5	Labrasol and Castor oil	Tween 80	Hot high-pressure homogenization technique	Various permeation enhancers are evaluated for skin penetration and retention. NLC with linoleic acid show better retention and sustain drug release than simple NLC	^61^
Cinnamon oil	For treatment of multi-drug resistant Pseudomonas aeruginosa burn wound infection	Precirol ATO 5	Labrafac lipophile WL 1349	Tween 80	High speed homogenization technique followed by sonication	Cinnamon oil loaded NLC show promising result in accelerated wound healing with antimicrobial property in rats in comparison to untreated mouse	^62^
Lidocaine and prilocaine	Topical anaesthesia and analgesia	Compritol 888 ATO, Precirol ATO 5 and glyceryl monostearate	--	Soya lecithin and Dimethyl dioctadecyl ammonium bromide	Solvent diffusion method	A dual drug is more effective than a single drug. Due to smaller particle size SLN show, high skin *ex vivo* penetration but NLC shows a better *in vivo* analgesic effect due to rapid drug release than SLN.	^63^
5-Fluorouracil	For the treatment of skin cancers and actinic keratosis	Precirol ATO 5	Labrasol	Poloxamer 188 and Solutol HS15	Hot homogenization technique	NLC loaded gel show high skin retention than non-NLC loaded gel. NLC shows initial burst release followed by sustained drug release.	^64^
Methotrexate	Treatment of psoriasis using NLC loaded gel	Compritol 888	Capmul MCM	--	High Speed homogenization technique	NLC gel shows the approximately 2-fold drug deposition in human cadaver skin than plain drug gel. Psoriatic area and inflammatory cytokines are reduced more rapidly than plain gel in imiquimod-induced psoriasis in mice.	^65^
Transcriptional transactivator peptide modified lidocaine	Transdermal delivery of drug for local anaesthesia	Soybean phospholipids	Labrafac PG	Cremophor ELP and tween 80	Solvent Evaporation Technique	Transdermal flux was higher than the plain drug and NLC. *In vivo* studies show anaesthesia enhanced transdermal delivery of drug by reducing the pain threshold in mice.	^66^

## Delivery through nasal route

 Conventionally, nasal route is used to deliver drugs for the treatment of local conditions such as nasal congestion, rhinitis, sinusitis, and allergic condition. High drug permeability, high blood flow, comparability low enzymes, and ability to bypass first-pass metabolism result in a faster and higher drug delivery rate through this route. Due to easily accessible sites with the ability to deliver the drug directly to the brain bypassing the blood-brain barrier result in increased attention towards these routes of drug delivery for the nose to brain drug targetting.^67^ Anatomically nasal cavity is divided into two chambers by the nasal septum. Among different regions of the nasal cavity respiratory and olfactory region is important for drug delivery. The respiratory region is rich in blood supply and drug delivery to the systemic circulation is indirectly achieved via the lungs.^68^ Olfactory region contains olfactory receptor neurons, supporting cells, and basal cells. Drug delivery to the brain can be possible through nerves or through supporting cells via a transcellular pathway. There is another trigeminal pathway through which drugs can reach the brain quickly.^69^

 Certain limitations hinder the drug delivery through this route. Enzymatic degradation of a certain drug in the nasal cavity along with solubility and permeability are common problems associated with different routes also. One of the major hurdles is low contact or residence time of the drug due to rapid inward and outward drug flow and mucociliary clearance of the drug in regular intervals. Along with this, the sensitive nature of nasal mucosa creates local irritation if the drug is not isotonic and has different pH.^70^

 NLC are nanosized biocompatible carriers that can deeply penetrate nasal mucosa and show sustained drug release. With the ability to deliver the drug in the nasal mucosa, these carriers are frequently investigated to deliver the drug into the brain. As NLC have several advantages over conventional liposome and SLNs make them suitable carriers. Along with the inherent advantages, good stability and ability to be incorporated into various gelling and bioadhesives make them more effective in nasal drug delivery.^69,71^ Management of various CNS-related conditions non-invasively such as glioblastoma, migraine, epilepsy, meningitis, etc along with quick delivery of the drug into the systemic circulation is possible through NLC.^31,71–74^ Table 3 shows nasal application of drug delivery by NLC.

**Table 3:** Drug Delivery through Nasal route by NLC

**Drug**	**Purpose**	**Solid lipid**	**Liquid lipid**	**Surfactant**	**Formulation method**	**Outcome**	**Ref.**
Almotriptan malate	Nasal delivery of antimigraine drug	Compritol	Labrafil	Tween 80 and lauroglycol	Hot homogenization and ultrasonication technique	In sheep nasal mucosa, chitosan-coated NLC showed enhanced mucoadhesion and high drug permeability, *in vivo* study shows high C_max_ than the solution and oral marketed formulation in albino rabbits	^72^
Lorazepam	Nasal delivery of drug for status epilepticus	Glyceryl monostearate	Oleic acid	Tween 80 and pluronic F127	Solvent diffusion and evaporation method	Chitosan-based NLC show a sustained *in vitro* drug release rate as well as better *in vivo* result in convulsion model rats.	^73^
Mosapride citrate	Nasal delivery of prokinetic agent for GERD	Stearic acid	isopropyl myristate	L-alpha lecithin and Lutrol F127	Melt–emulsification low temperature–solidification technique	Drug loaded NLC show 3-fold increase in drug permeation in sheep nasal mucosa and increased gastric emptying rate and bioavailability in comparison to a drug suspension and marketed oral preparation.	^74^
Oleuropein	Phytochemical loaded NLC for treatment of meningitis	Tefose	Capmul	Poloxamer 188, polysorbate 80, and soy lecithin	Melt emulsification and ultrasonication method	High permeation of drugs is seen in the nasal mucosa. Initial burst release followed by sustained release is shown in *in vitro* studies.	^31^
Carbamazepine	For fast action of anti-epileptic drug through nose-to-brain targeting	Precirol ATO 5	Capmul MCM	Tween 80 and span 20	Microemulsion technique followed by probe sonication	Thermosensitive *in situ* gel using poloxamer 407 (P407), poloxamer 188 (P188), and the mucoadhesive polymer were used. NLC-based gel exhibits greater drug diffusion in sheep nasal mucosa and anticonvulsant activity in MES model rats than non-NLC-based gel and drug dispersion.	^75^
Flibanserin	Nose-to-brain delivery of seratonergic agent	Glyceryl behenate	Sweet almond oil	L-phosphatidyl choline and gelucire 44/14	High speed homogenization followed by sonication	Optimized formulation shows better drug release. *In vivo* assessment shows high plasma and brain drug concentration than raw drug.	^76^
Escitalopram and Paroxetine	Nose to brain drug delivery for treatment of depression	Precirol ATO 5	Lauroglycol 90	Tween 80	High Pressure Homogenization Technique	*In vivo* assays of NLC shows similar systemic drug with respect to i.v. drug administration. Borneal encapsulation result in five-time greater brain drug concentration with less systemic exposure.	^77^

## Delivery through parenteral route

 The Parenteral Route is the most effective method of delivering drugs directly into the systemic circulation. It is the preferred route for the drug with a narrow therapeutic index and poor bioavailability. For emergency treatment of unconscious patients, the parenteral route is the only choice. One of the most discouraging characteristics of the parenteral route is injection of drug cause pain and discomfort with the need for assistance.^78^ NLC has several advantages that make it a suitable carrier system for drug delivery. NLC can encapsulate water-insoluble lipophilic drugs to deliver them to the desired size. NLC showed a sustained drug release profile which reduces the injection frequency.^79,80^ With surface modification, it is possible to target several organs and tumor cells. Which helps develop a safe and effective formulation of drugs with high toxicity and low therapeutic index. Employment of NLC is suitable for treating various carcinomas, infections, and diseases that involve targeting drugs to the brain such as neurodegenerative diseases.^80–84^ Table 4 shows examples of NLC system which are delivered specifically by the parenteral route.

**Table 4:** Drug delivery through parenteral route by NLC

**Drug**	**Purpose**	**Solid lipid**	**Liquid lipid**	**Surfactant**	**Formulation method**	**Outcome**	**Ref.**
Miltefosine	Prolong drug release of anticancer drug	Stearic acid	Oleic acid	Tween 80	Microemulsion technique	*In vitro *and *in vivo *studies in tumor bearing mice show sustained drug release with increased blood circulation and reduction in clearance rate as well as increased hemolytic properties with respect to free drug in mouse.	^79^
Carvacrol	Anti-leishmanial NLC for increasing systemic residence time with less toxicity	Stearic acid	Beeswax	Poloxamer 188	Microemulsion technique	*In vitro *results show initial burst release followed by sustained drug release unlike free drug which show quick release. *In vivo* results show increased half-life and low clearance of drug loaded in NLC with less cytotoxicity.	^80^
Doxorubicin	Delivery of pH sensitive anticancer drug coupled with tocopherol succinate through amide and hydrazone conjugate	Compritol	Docosahexaenoic acid	Tween 80	Emulsion ultrasonication technique	pH sensitive hydrazone bond shows controlled release from NLC. *In vivo *studies show better pharmacokinetic profile and reduced tumor growth with decrease in drug induced toxicity in mice.	^84^
Resveratrol	RBC membrane coated NLC containing rabies virus glycoprotein and triphenylphosphine cation molecule for antioxidant delivery to neuronal mitochondria for Alzheimer's disease	Cetyl palmitate	Oleic acid and cholesterol	Tween 80	Modified emulsification ultrasonication method	RBC encapsulation result in higher biocompatibility and sustained drug release. *in vivo *studies in mice shows dual modified NLC can cross BBB and deliver antioxidant to specifically to neuronal mitochondria.	^81^
Sesamol	Delivery of antioxidant and neuroprotective agents to patients suffering from ischemic stroke	Cetyl palmitate	Oleic acid	Polaxomer 188 and Tween 80	High pressure homogenization	Sesamol loaded intravenous injection of NLC show reduced cytotoxicity, cellular damage and oxidative stress in rats, while free Sesamol failed.	^82^

## Delivery through ophthalmic route

 The eye is one of the most sensitive and challenging organs for drug delivery. Drugs with low bioavailability or potential systemic toxicity which are needed for chronic ocular diseases like diabetic retinopathy, ocular infections, or other conditions which need long-term therapy are preferred for ocular drug delivery. Conventional ocular therapy needs frequent dosing which leads to patient discomfort.^85^ Systemic administration of drugs for ophthalmic use is not preferred due to the blood ocular barrier. As the eye is exposed to the outer environment and is a sensitive organ several barriers make ocular delivery of drugs the most challenging task for formulators.^86^

 Anatomically eye is divided into two segments: The first anterior segment covering one-third part of the eye consists of cornea and lens assembly and the Second posterior segment covering a two-third portion of the eye consist of the retina and vitreous humor. There is a precorneal barrier, static barrier, and dynamic barrier due to the cornea, sclera, and retina making a blood-aqueous barrier and blood-retinal barrier.^87^ Along with that surface removal of the drug due to lachrymal fluid secretion in the eye as well as blinking makes drug delivery difficult. Epithelial barrier on the outer corneal layer limits entry of hydrophilic drugs and macromolecules.^88^

 NLC is a promising carrier for ocular administration because of its biocompatible nature. Penetration in the eye is favored due to the small size, non-toxic, non-immunogenic, and lipidic nature of NLCs.^89^ NLC formulation for ocular delivery contains a non-ionic surfactant. This formulation protects the drug from chemical degradation. Mucoadhesion along with small particle size act as a drug depot in the eye resulting in improved trans corneal diffusion and cellular uptake. Depot formation of NLC show sustained release of drug with less frequent administration.^90^ Table 5 shows the employment of NLC based system for the delivery of drugs through the ophthalmic route.

**Table 5:** Drug delivery through ophthalmic route by NLC

**Drug**	**Purpose**	**Solid lipid**	**Liquid lipid**	**Surfactant**	**Formulation method**	**Outcome**	**Ref.**
Nepafenac	Treatment of inflammation following cataract surgery	Glycerine monostearate	Miglyol 812N	Cremophor EL and soy lecithin	High speed homogenization followed by ultra-probe sonication	NLC loaded shows high gelation temperature than normal hydrogel. Sustained *in vitro* drug release is obtained by NLC loaded gel with no cytotoxicity in human corneal epithelial cells and high cellular penetration.	^83^
Ciprofloxacin	Antibacterial agent loaded NLC for treatment of Bacterial Endophthalmitis	Precirol ATO 5	Oleic acid	Tween 80 and poloxamer 188	High speed homogenization technique	Gellan gum is used as a gelling agent in drug-loaded NLC *in situ* gel. Transcorneal permeability and flux is improved *in vitro* studies. NLC show higher residence time which results in sustained drug release.	^91^
Baicalin	pH and temperature sensitivity ophthalmic gel	Compritol 888 ATO	Miglyol 812N	Cremophor EL and soy lecithin	Melt emulsification and ultra-sonication technique	The combination of carboxymethyl chitosan and poloxamer 407 (F127) crosslinked by genipin is used for dual pH and thermosensitive applications. semi Interpenetrating polymer networks hydrogel show high corneal permeation due to precorneal retention of baicalin than normal eye drops.	^30^
Dexamethasone	Treatment of dry eye disease	Cholesterol	Labrafac lipophile WL1349	Tween 80	Solvent diffusion technique	NLC shows cellular internalization in human corneal epithelial cells and corneal distribution in the porcine cornea. In *ex vivo *study ELISA show 5-fold reduction in TNF α production with reference to dexamethasone solution.	^92^
Palmitoylethanolamide	Treatment of diabetic retinopathy	Compritol 888 ATO	Miglyol 812	Lutrol F68	High shear homogenization	Drug-loaded NLC shows the high retinal distribution in rat eye following a single instillation. Topical administration of NLC inhibits retinal TNF α in streptozotocin-induced diabetic rats.	^93^

## NLC as a gene delivery system

 Development in the field of medicine, biotechnology, and genetic engineering results in a better understanding of complex diseases and their treatment, which is not possible with the conventional approach. Delivery of RNA and DNA is explored for the treatment and immunization of genetic or acquired diseases. Direct transfer of genetic material in the cell is a difficult task because of the susceptibility of degradation, hydrophilic nature, and negative charge of this large size molecule. This challenging task of delivering genetic material can be accompanied by the utilization of vectors for gene delivery into cells.^94^ Viral vectors are frequently used for gene transfection but due to the risk associated with a viral vector, a recently wide range of non-viral vectors are being explored.

 Non-viral vectors show less immunogenicity, better flexibility, and low cytotoxicity. Target-specific gene delivery can be accomplished by non-viral surface and structural modification. Gold nanoparticles, carbon nanotubes, polymeric nanoparticles, liposomes, dendrimers, and SLNs are widely investigated for gene delivery. Lipid-based nanoparticles proved to better alternative due to better biocompatibility.^95^ NLC being one of the lipid carriers with inherent advantages such as stability and better drug loading capacity than its counterpart draws the attention of researchers for gene delivery.

 NLC can be incorporated into different formulations for the delivery of genetic material through different routes due to its relatively stable nature than other nano vectors.^96^ Apart from systemic delivery of gene inhalation and transdermal route is also explored for the site-specific delivery and reduced toxicity as in lung cancer.^97,98^ Mainly cationic lipid or positively charged nanocarriers are utilized but due to inherent cytotoxicity or nanotoxicity, thus further investigations are needed to make NLC an effective gene carrier.^99^ Neutral NLC tends to show lower toxicity than cationic NLC.^100^

 A wide range of diseases like cancer and multi-drug resistant cancer, severe infections, AIDS, Alzheimer’s and Parkinson’s disease can be treated with the use of gene therapy.^101^ Gene therapy not only involves the delivery of DNA but also RNA for expression in a host cell, small interfering RNA (siRNA), messenger RNA (mRNA), and microRNA (miRNA) are the types of genes that are used in gene therapy. With the approval, of the COVID-19 vaccine based on lipid nanoparticles with enclosed mRNA, the gene delivery system is also being explored for the new future mRNA-based vaccine.^102^ Gene therapy and vaccination are different domains but due to the delivery of RNA in a host cell is involved we have summarized both in one section. Table 6 gives a brief account of the utilization of NLC for gene delivery.

**Table 6:** NLC as a gene delivery system

**Gene loaded**	**Drug loaded**	**Route of administration**	**Purpose**	**Outcome**	**Ref.**
EGFR-specific siRNA duplexes	Paclitaxel	Inhalation and i.v.	Treatment of lung cancer with chemo and gene therapy	Effective transfer of siRNA and drug is seen in cancer cells. *In vivo *studies show inhalation results in higher distribution in lungs while iv administration failed to reach lung with tumor.	^97^
Enhanced green fluorescence protein pDNA	Temozolomide	i.v.	Gliomatosis cerebri combination therapy	A 4-fold decrease in IC_50 _value was seen with higher gene transfection efficiency. *In vivo *results show 3-time higher tumor inhibition by co loaded NLC than plain drug.	^103^
Fluorescent AF488- 231 siRNA	-	Topical	Localized delivery for wound healing	Collagen scaffold loaded with gene encapsulated NLC show siRNA delivery at sustained rate and downregulation ERK-1 protein was seen	^98^
Zika virus antigens encoding rvRNA	-	i.m.	Single low dose vaccine for zika virus	A stable two-vialed vaccine is prepared and optimized to provide immunogenicity in lowest dose. NLC loaded rvRNA provides protection against zika virus in mice.	^104^
TNF-α siRNA	Tacrolimus	Topical	Treatment of Psoriasis	Cellular transfection studies show presence of RNA in cytosol. 7-fold reduction of cytokine TNF-α expression in mice is shown in *in vivo *studies.	^105^
siRNAs (MMP3, CCL12, and HIF1A)	Prostaglandin E	Inhalation	Treatment of idiopathic pulmonary fibrosis	*In vivo *studies show inhalation in animal model of mice results in reduced body mass which is indicator of reduction in pulmonary fibrosis. Lung delivery is achieved with low tissue damage and mortality is seen.	^106^
Plasmid-enhanced green fluorescent protein pDNA	Paclitaxel	i.v.	Targeted lung cancer therapy	Cationic surface modified NLC with transferrin was prepared. High gene transfection and enhanced antitumor activity with low cytotoxicity is confirmed in *in vitro* and *in vivo *studies.	^107^

## Conclusion

 NLCs are made possible by combining nanotechnology with lipids as a structural material. NLC are a versatile platform for drug delivery via various routes. These nanocarriers have drawn the interest of researchers from all over the world. Partially crystallized lipidic nanocarriers have high drug loading and stability than their predecessors such as liposomes, nanoemulsions, and SLNs. These nanocarriers are biodegradable, biocompatible, safe, and effective with high drug loading capacity. These nanocarriers can also be employed for active or passive drug targeting to different organs or tumor cells. NLC has proven its worth in the cosmetics industry, but its potential in the pharmaceutical industry has yet to be fully explored. NLC can be useful in diseases whose management is difficult, such as cancer, infections, neurodegenerative diseases, localized drug delivery as well as genetic diseases which conventional carrier systems cannot achieve. More research is required in this area to shift these interesting nanocarriers from lab to market. There is a need to evaluate the toxicity profile of such nanocarriers and solve the problems associated with nanomedicines such as complexity in formulation and characterization. Lack of sufficient clinical studies and data results in a slow development of these nanocarriers.

## Competing Interests

 None.

## Ethical Approval

 Not applicable.
